# Correction: Regulation of retinal axon growth by secreted Vax1 homeodomain protein

**DOI:** 10.7554/eLife.05657

**Published:** 2014-11-24

**Authors:** Namsuk Kim, Kwang Wook Min, Kyung Hwa Kang, Eun Jung Lee, Hyoung-Tai Kim, Kyunghwan Moon, Jiheon Choi, Dai Le, Sang-Hee Lee, Jin Woo Kim

Kim N, Min KW, Kang KH, Lee EJ, Kim HT, Moon K, Choi J, Le D, Lee SH. 2014. Regulation of retinal axon growth by secreted Vax1 homeodomain protein. *eLife* **3**:e02671. doi: http://dx.doi.org/10.7554/eLife.02671

Published 8 September 2014

In the published article there was a typographical error in Figure 9B. The fourth and fifth labels on the x-axis were wrongly given as ‘6X-His + Robo-Fc’ and ‘Vax1 + Robo3-Fc’. These labels should be ‘6X-His + Robo1-Fc’ and ‘Vax1 + Robo1-Fc’ respectively.

This article has been corrected accordingly.

